# Holiday Souvenir Gone Wrong: Laser Pointer-Induced Maculopathy

**DOI:** 10.7759/cureus.109727

**Published:** 2026-05-27

**Authors:** Mohamed-Amine Ouacha, Sandrine De Temmerman

**Affiliations:** 1 Ophthalmology, Hôpital de Jolimont, La Louvière, BEL; 2 Ophthalmology, Cliniques Universitaires Saint-Luc, Bruxelles, BEL; 3 Ophthalmology, Chu Helora, Site Jolimont, La Louviére, BEL

**Keywords:** green laser, laser induced maculopathy, oct (optical coherence tomography), optical coherence tomography, outer retinal ellipsoid zone disruption, pediatric retinal injury

## Abstract

Self‑induced maculopathy caused by handheld laser pointers has emerged as an increasingly recognized source of retinal injury in children and adolescents. We report the case of a 13‑year‑old girl with pre‑existing unilateral amblyopia and small‑angle strabismus who presented with a paracentral scotoma and bilateral visual reduction following exposure to a non‑certified 100 mW green laser pointer purchased during a holiday in Spain. On presentation, best‑corrected visual acuity measured 8/10 in the right eye and 7/10 in the left eye. Fundus examination revealed a central macular retinal pigment epithelium lesion in the right eye. Optical coherence tomography demonstrated disruption of the ellipsoid zone at the right foveola, consistent with laser‑induced maculopathy. The patient was managed conservatively with observation and strict avoidance of further laser exposure. At three months, right‑eye visual acuity improved to 10/10, indicating partial yet incomplete recovery. This case highlights that laser‑pointer‑induced maculopathy may present asymmetrically with relatively mild visual impairment and a generally favorable prognosis, while underscoring the need for public awareness and stronger regulation of high‑power laser devices marketed as toys.

## Introduction

Laser pointers are widely available and often mistakenly regarded as harmless toys. High-power devices can seriously damage the macula if aimed at the eyes, particularly in children who may not recognize the danger [1]. Non-certified green laser pointers, frequently sold online or in informal settings, may greatly exceed the power limits considered safe for recreational use [2]. Recent reports have described an increasing number of laser pointer-induced maculopathy cases, most often in pediatric patients and in individuals with pre-existing visual vulnerability, with variable visual outcomes depending on laser power, exposure duration, and promptness of diagnosis. Children with pre-existing visual impairment, such as amblyopia, are particularly vulnerable to additional functional loss. Despite these reports, important knowledge gaps remain regarding long-term prognosis and the optimal management of mild, uncomplicated cases, especially in children with pre-existing visual impairment such as amblyopia.

We describe a 13-year-old girl with unilateral amblyopia who developed unilateral laser-induced maculopathy after exposure to a high-power green laser pointer. We highlight the clinical and optical coherence tomography (OCT) findings, the clinical course, and the preventive implications, emphasizing both the particular vulnerability of amblyopic patients and the public health importance of regulating high-power laser pointers.

## Case presentation

A 13-year-old Belgian girl was followed in a pediatric ophthalmology clinic for stable unilateral amblyopia associated with small-angle strabismus. Her previous best-corrected visual acuity was 10/10 in the right eye (RE) and 7/10 in the left eye (LE).

She presented urgently with recent visual deterioration and a paracentral scotoma that appeared immediately after playing with a laser pointer she had received during a holiday in Spain. The device was described as a non-certified green laser pointer manufactured in China, with a stated output power of 100 mW and classified as Class IIIb. The patient admitted to repeatedly directing the laser beam toward a mirror, causing multiple reflections toward her face and eyes, but she and her parents were unable to provide a reliable estimate of the exact duration or number of exposures. No immediate pain or discomfort was reported.

She complained of a paracentral scotoma and central visual blur in the RE, accompanied by difficulty reading. There was no redness, ocular pain, history of trauma, recent infection, or medication use.

Anterior segment examination was entirely normal in both eyes. At presentation, best-corrected visual acuity measured 8/10 in the RE and 7/10 in the LE. Previously, the RE had consistently reached 10/10, while the LE remained amblyopic but stable. Ocular motility was normal, with a small-angle strabismus of 6 prism diopters and right-eye dominance.

**Figure 1:**
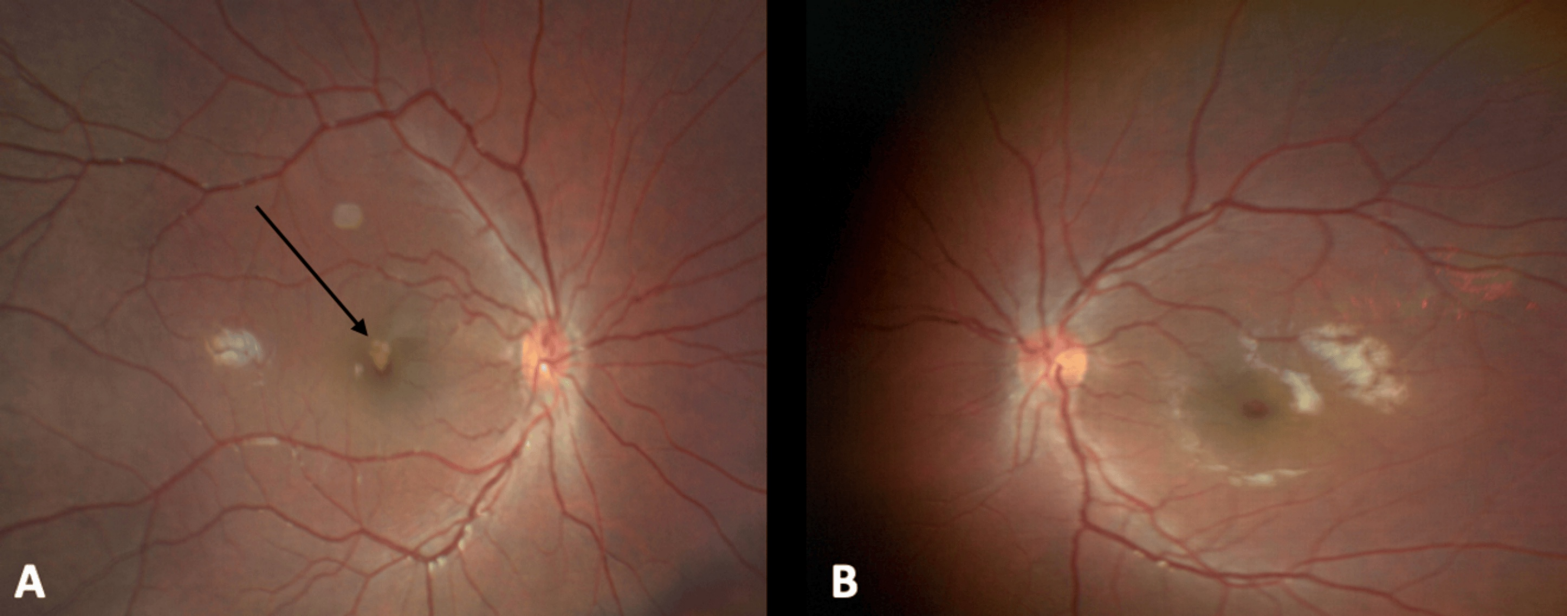
Color fundus photographs of both eyes at presentation. The right eye shows a subtle foveolar lesion compatible with laser-induced maculopathy. (A) Right eye. (B) Left eye. Arrow: Foveolar lesion in the right eye.

Dilated fundus examination revealed a small, well-defined yellowish lesion approximately 0.45 mm in size centered on the fovea in the RE, as illustrated in Figure 1. In the LE, similar but less pronounced yellowish changes were observed in the posterior pole. The appearance of the lesions, sharply demarcated pigmentary alterations without hemorrhage, exudates, or signs of vasculitis or chorioretinitis, favored a phototoxic origin rather than inflammatory or vascular pathology.

**Figure 2:**
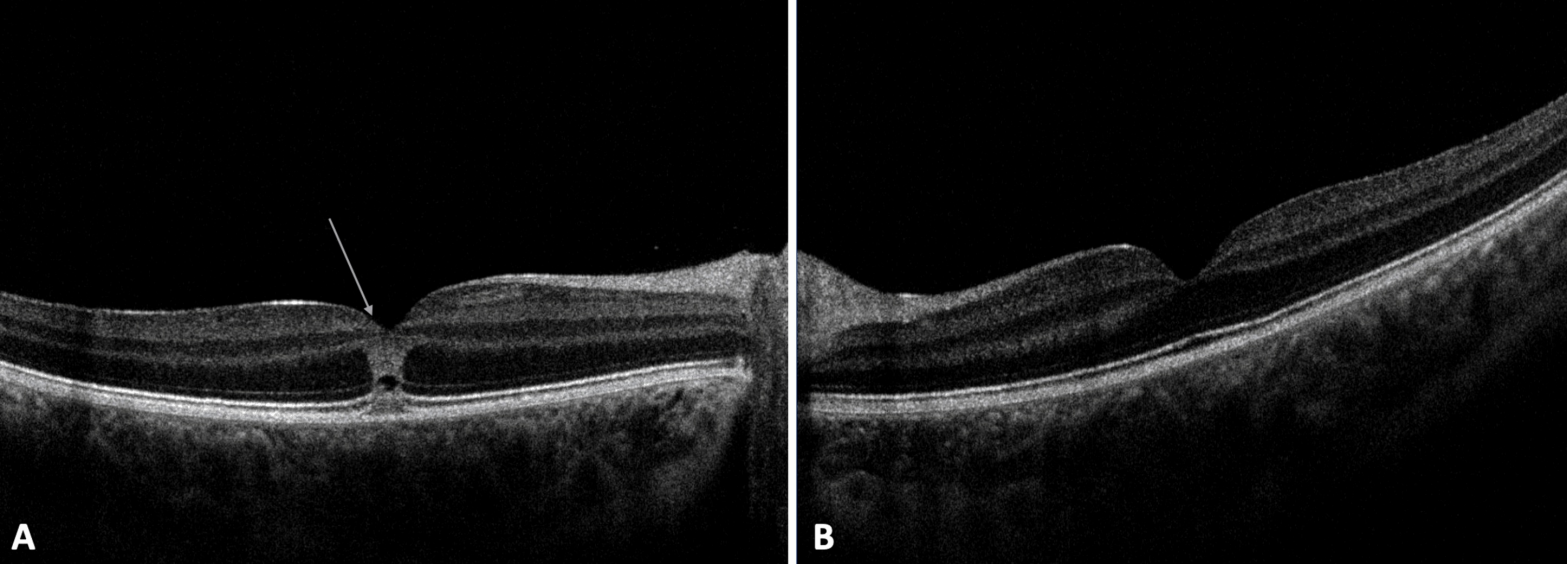
Spectral domain OCT scans of both maculae at presentation. The right eye shows a hyperreflective band involving the outer retina at the foveola with clear disruption of the ellipsoid zone, while the left eye displays a normal macular profile. (A) Right eye. (B) Left eye. Arrow: Disruption of the architecture of the outer retina at the fovea. OCT: optical coherence tomography.

Spectral-domain OCT of the right macula showed structural alterations, as illustrated in Figure 2. Initial scans demonstrated a hyperreflective band extending from the retinal pigment epithelium (RPE) to the outer plexiform layer, with irregularities of the outer retina and RPE at the foveola. A subsequent scan clearly revealed disruption of the ellipsoid zone, a pattern characteristic of laser-induced maculopathy described in the literature.

Given the recent exposure to a high-power green laser pointer, the foveal location of the lesions, and the typical OCT findings, a diagnosis of laser-induced maculopathy was established. In the absence of signs of active inflammation or neovascular complications, conservative management was adopted. The patient and her parents were instructed to strictly avoid further exposure to laser devices.

No systemic or topical treatment was initiated. Regular follow-up visits were scheduled to monitor visual acuity and retinal structural changes.

At the one-month follow-up, the patient reported subjective improvement with a reduction of the paracentral scotoma. Best-corrected visual acuity improved to 9/10 in the RE and remained 7/10 in the LE. OCT showed decreased hyperreflectivity and thickening of the outer retina in the foveal zone, with persistent ellipsoid zone disruption.

At the three-month follow-up, visual acuity improved further to 10/10 in the RE and remained stable at 7/10 in the amblyopic LE. OCT demonstrated partial resolution of the outer retinal abnormalities with incomplete restoration of the ellipsoid zone, consistent with partial recovery described in laser-induced and solar-induced maculopathies. No secondary complications, such as choroidal neovascularization, were identified.

**Figure 3:**
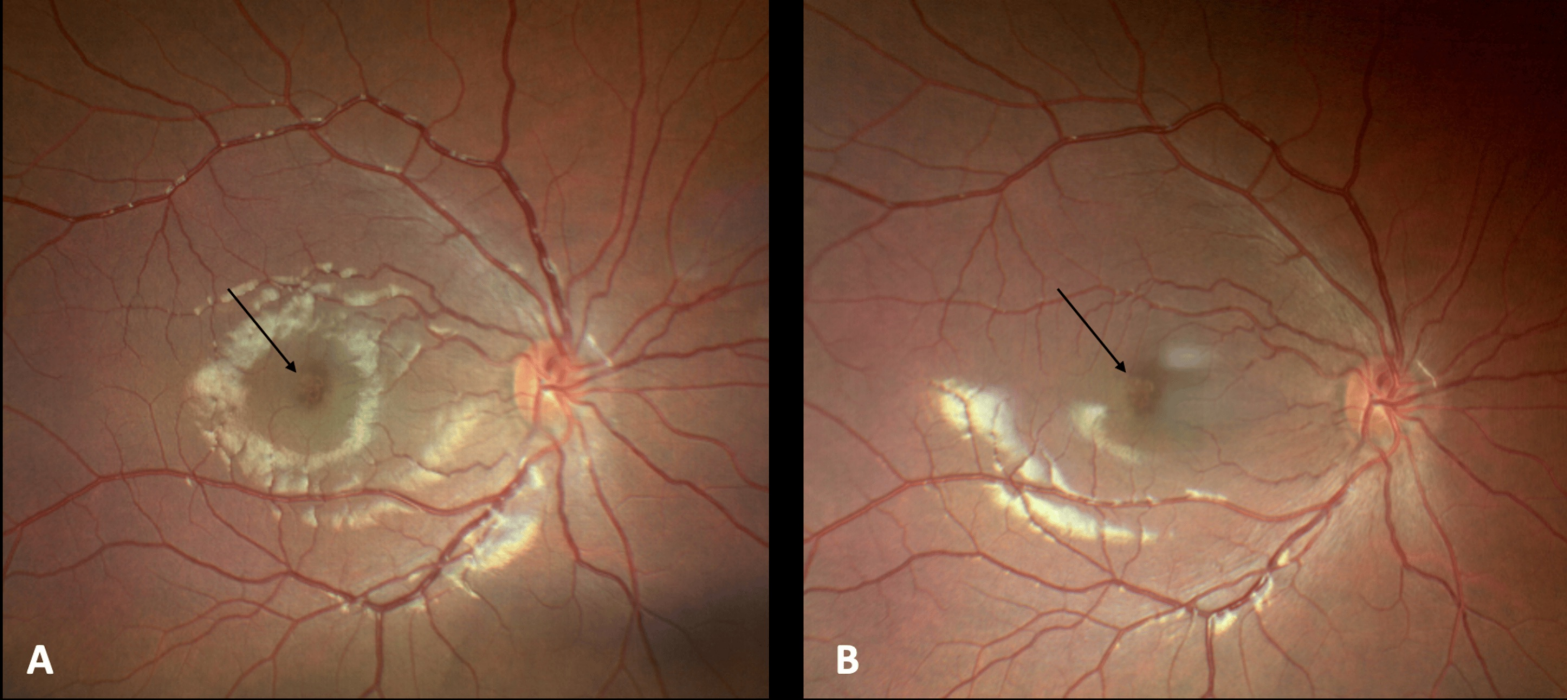
Color fundus photographs of the right eye at one-month and three-month follow-up showing progressive fading of the foveolar lesion and restoration of a near normal macular reflex (A) One-month follow-up. (B) Three-month follow-up. Arrows: Foveolar lesion.

**Figure 4:**
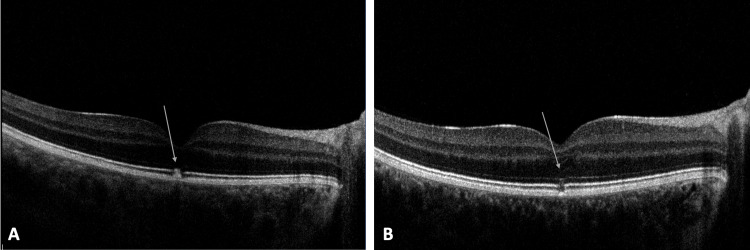
Spectral domain OCT of the right macula at one-month and three-month follow-up, demonstrating initial focal thickening and remodeling of the outer retina at the foveola, followed by anatomical improvement with partial restoration of the ellipsoid zone (A) One-month follow-up. (B) Three-month follow-up. Arrows: Foveolar lesion. OCT: Optical coherence tomography.

The OCT and fundus evolution at one-month and three-month follow-up are illustrated in Figures 3, 4. 

Written informed consent was obtained from the patient’s legal guardians for publication of this case report and accompanying images.

## Discussion

This case illustrates asymmetrical macular damage from a high-power green laser pointer in a child, involving the better-seeing eye in the context of pre-existing amblyopia and strabismus. Laser-induced retinal injury is thought to result from photothermal and/or photomechanical effects, producing focal damage to the RPE and photoreceptors at the fovea, where incoming light is concentrated. Green laser pointers usually operate at a wavelength around 532 nm, which is more strongly absorbed by RPE melanin and hemoglobin, thereby increasing the likelihood of photothermal damage to the outer retina and RPE compared with longer-wavelength red devices. A laser device with an output power between 5 mW and 500 mW should be classified as Class IIIb (3B), and pointers with outputs around 100 mW clearly exceed the limits considered safe for direct ocular exposure and should not be regarded as toys [3,4].

Laser-induced maculopathy typically presents with sudden-onset decreased central vision, metamorphopsia, or scotoma. In children, the history may be incomplete or initially denied, and parents may underestimate the danger of laser pointers marketed as toys [5]. Careful questioning about recent exposure to lasers, particularly green lasers, is therefore crucial when evaluating unexplained macular changes in this age group [6].

Numerous case reports and small series describe similar macular burns in children and adolescents exposed to “toy” or recreational laser pointers, frequently purchased online or through informal channels, with actual power ratings far above those advertised [7]. Bilateral involvement is common, as users tend to look directly into the beam or repeatedly expose both eyes [8,9]. Our case shares several typical characteristics with the published literature, including the pediatric age, use of a high-power green pointer, and OCT evidence of outer retinal damage, but also presents some particularities. The asymmetry of the lesions and the more subtle posterior pole changes in the LE may be related to the pre-existing strabismus, with non-central fixation and functional neutralization of the amblyopic eye. The presence of amblyopia increases the functional impact of any additional macular damage in the better-seeing eye, even when anatomical changes appear relatively limited. This underscores the particular vulnerability of patients with pre-existing visual impairment.

OCT is central to diagnosis and monitoring. Typical findings include initial outer retinal hyperreflectivity, followed by disruption of the ellipsoid zone, damage to the external limiting membrane, and RPE irregularities, sometimes accompanied by foveal excavation or microcavitation [10-12]. Several studies have documented partial recovery of the ellipsoid zone over time, correlating with functional improvement, although structural and functional outcomes vary widely. In our case, right-eye acuity improved from 8/10 to 10/10 at three months, with persistent but attenuated outer retinal changes.

There is no consensus on optimal treatment. Reported approaches range from observation alone to topical or systemic corticosteroids, intravitreal corticosteroids, and anti-VEGF therapy in cases complicated by choroidal neovascularization [13,14]. Robust evidence of benefit is lacking, and prognosis appears to depend mainly on the severity of initial damage and the potential for foveal recovery rather than on a specific treatment regimen. In our patient, we chose close observation without pharmacological treatment, combined with clear education on the risks associated with laser pointers. The good functional and anatomical outcome supports the appropriateness of the conservative approach in mild cases without complications.

From a public health perspective, this case reinforces the need for strict prevention. High-power laser pointers should not be marketed or used as children’s toys. Families, teachers, pediatricians, and ophthalmologists must be made aware that even brief exposure can cause significant macular injury and permanent visual loss. Regulatory measures limiting the sale and labeling of high-power laser devices, particularly non-certified imports, are crucial to reducing these preventable injuries [15].

## Conclusions

Laser pointer-induced maculopathy is a preventable cause of visual loss in children. In this 13-year-old amblyopic girl, exposure to a non-certified Class IIIb (3B) 100 mW green laser pointer caused macular damage with ellipsoid zone disruption on OCT in the RE, while only subtle posterior pole changes were observed in the left eye. Although visual acuity in the RE improved over three months, structural sequelae persisted on OCT, illustrating that functional recovery may occur despite residual outer retinal alterations. This single case suggests that a conservative approach may be appropriate in mild, uncomplicated presentations, but outcomes are likely to vary according to laser power, exposure duration, and baseline ocular status. Primary prevention through public education and stricter regulation of high-power laser pointers remains essential to reduce these avoidable injuries.
